# Mucinous cystic neoplasms of the liver with biliary prolapse

**DOI:** 10.1007/s11604-022-01361-3

**Published:** 2022-11-19

**Authors:** Kazuto Kozaka, Hiroaki Takahashi, Akitoshi Inoue, Rondell P. D. Graham, James H. Boyum, Jay P. Heiken, Naoki Takahashi

**Affiliations:** 1 Department of Radiology, Mayo Clinic College of Medicine, Mayo Clinic, 200, First Street SW, Rochester, MN 55905 USA; 2 Department of Radiology, Kanazawa University Graduate School of Medical Sciences, Kanazawa, Japan; 3 Department of Pathology, Mayo Clinic College of Medicine, Mayo Clinic, 200, First Street SW, Rochester, MN 55905 USA

**Keywords:** Liver neoplasms, Magnetic resonance imaging, Tomography, X-Ray computed, Bile ducts, Intrahepatic

## Abstract

**Objectives:**

To describe the prevalence, clinical and radiological findings of biliary prolapse in pathologically proven mucinous cystic neoplasm of the liver (MCN-L).

**Methods:**

Thirty-four patients, all female with median age 50 years (range, 14–82), with histologically confirmed MCN-L were enrolled. Median tumor size was 9 cm (range, 2–21 cm). Fifty-seven examinations (17 ultrasound, 25 CT, and 15 MR) among 34 MCN-Ls were reviewed. Two radiologists retrospectively assessed images for tumor location, size and other morphological features of the tumor, presence of biliary prolapse and upstream bile duct dilatation. Ultrasound, CT, and MR were assessed separately. Clinical features were evaluated. Clinical and radiological characteristics of MCN-L with and without biliary prolapse were compared.

**Results:**

15% (5/34) of MCN-Ls showed biliary prolapse confirmed at pathology. None of MCN-Ls were associated with invasive carcinoma. Patients with biliary prolapse were significantly younger than those without (median 27 years [22–56] vs. median 51 years [14–82], *p* = 0.03). MCN-Ls with biliary prolapse were significantly smaller than those without (median 6.4 cm [2.2–7.5] vs. median 9.6 cm [3.1–21], *p* = 0.01). The upstream bile duct was dilated more frequently in MCN-Ls with biliary prolapse (100% vs. 38%, *p* = 0.02). Jaundice was significantly more common in MCN-Ls with biliary prolapse (80 vs 3%, *p* = 0.0005). Other clinical or radiological features were not significantly different between two groups.

**Conclusions:**

Biliary prolapse was found in 15% of MCN-Ls. MCN-Ls with biliary prolapse were significantly smaller and were more commonly associated with upstream bile duct dilation and jaundice than those without biliary prolapse.

**Supplementary Information:**

The online version contains supplementary material available at 10.1007/s11604-022-01361-3.

## Introduction

Mucinous cystic neoplasm of the liver (MCN-L) is a distinct type of cystic biliary tumor, which was proposed in the World Health Organization (WHO) classification in 2010 [1] and again in 2019 [2]. The histologic definition of MCN-L is that of a cyst-forming epithelial neoplasm composed of a single layer of cuboidal or tall columnar mucinous epithelial cells overlying a stroma of spindle cells that resemble ovarian-like stroma (OLS). The presence of OLS is required for pathologic diagnosis of MCN-L, and the terms hepatobiliary cystadenoma and cystadenocarcinoma are not recommended to use especially when OLS is presented in the wall of hepatic cystic tumor [2]. Almost all MCN-Ls occur in women and majority in middle age [2–5]. Some of the atypical cases initially called hepatobiliary cystadenoma/adenocarcinoma, such as those occurring in men, tumors communicating with bile ducts, and cystic tumors without OLS [2, 6, 7] are now considered more likely to be other cystic liver tumors, including intraductal papillary neoplasm of the bile duct (IPNB) [2, 8].

Since the only treatment for MCN-L is surgical resection, it is clinically significant to accurately diagnose and distinguish MCN-L from other cystic liver lesions such as simple bile duct cysts, cystic IPNB, and cystic metastases. Characteristic imaging features of MCN-L have been reported including the presence of a thick capsule, multiple loculations formed by thick internal septa, lack of biliary communication, and typical location in the left hepatic lobe [4, 5, 9, 10]. Takano et al. also reported that bile duct prolapse and expansive growth appear to be characteristic behavior of MCN-L [11]. Indeed, previous case reports or small case series have shown that the presence of biliary prolapse serves as an important diagnostic clue [11–13]. To date, only 16 cases of biliary prolapse of MCN-L have been reported in the radiology literature [11–13], and the frequency of this finding and related clinical and imaging features has never been elucidated in large case series. Therefore, the aim of the study is to describe the prevalence, clinical, and imaging findings of MCN-L with biliary prolapse.

## Materials and methods

### Subjects

This is an institutional review board approved, HIPPA-compliant retrospective study, and the need to obtain informed consent was waived. Sixty-seven cystic liver neoplasms were identified by electronically searching our institutional pathology database from January 1998 to September 2019, using keywords: “mucinous cystic neoplasm”, “biliary cystadenoma”, or “biliary cystadenocarcinoma”. A total of 67 cases were evaluated by a board-certified pathologist (R.P.G.). Diagnosis of MCN-L was confirmed in 34 cases according to the WHO classification published in 2019 [2] and included in the study. Due to lack of OLS, 33 cases were excluded. All 34 patients were female with a median age of 50 years (range 14–82) and had at least one of US, CT, or MRI examination within one year prior to surgery.

### Image acquisition

CT, US, and MRI examinations were performed in 25/34 (74%), 17/34 (50%), and 15/34 (44%) patients, respectively. The median time between the imaging and surgery was 34 days (range 6–352) for ultrasound, 44 days (range 1–302) for CT and 42 days (range 7–297) for MRI. Eighteen patients had one imaging modality (US, *n* = 4; CT, *n* = 11; MRI, *n* = 3), 9 had two imaging modalities (US + CT, *n* = 4; US + MRI, *n* = 2; CT + MRI, *n* = 3), and 7 had all three imaging modalities. On US examination, because of retrospective design of this study, only captured still images were available to assess. On CT examination, precontrast, postcontrast, both precontrast and postcontrast images were available in 1, 19, and 4, respectively. On MR examination, all had T2 weighted, precontrast T1 weighted images and twelve had MR cholangiopancreatography (MRCP) including one 2-dimensional and 11 3-dimensional sequences. Twelve had multiphasic dynamic contrast-enhanced T1 weighted images. Fifteen patients had both pre- and postcontrast CT and/or MRI.

### Image analysis

Fifty-seven examinations (25 CT, 17 US, and 15 MR) were independently reviewed by two board-certified abdominal radiologists (A.I. and H.T. with 13 and 8 years of experiences in abdominal radiology) who were blinded to the clinical information and other imaging tests. A reading session was held for each modality of examinations, spaced at least 2 weeks interval to avoid recall bias.

The reviewers were instructed to pay particular attention to the relationship between the cystic tumor and the bile ducts. Biliary features assessed included upstream and downstream bile duct dilation, communication between the cystic tumor and the biliary tract, and biliary prolapse of the tumor. Biliary prolapse is considered present when the cystic mass extends into bile duct with cyst wall or septation visualized within the duct. Other image features evaluated were selected from reports of cystic liver neoplasms published in the radiology literature [3, 4, 14–18]. The morphologic features evaluated included the presence of lobulation, cyst wall thickness > 2 mm being considered thick, cyst wall irregularity, cyst wall hemorrhage assessed by T1 weighted images, cyst wall or septal calcification assessed by US or precontrast CT, the presence of septa, and cyst-in-cyst appearance (defined as a cyst smaller than one fourth the diameter of the entire lesion attached to the cyst wall or a septum without external indentation). The reviewers also evaluated enhancement of the cyst wall or septa and the presence of an enhancing solid portion within the cyst by both pre- and postcontrast CT and/or MRI. The cyst fluid attenuation/signal intensity was rated negative (anechoic on US, iso to cerebral spinal fluid on CT and MRI) or positive (non-anechoic on US; high-/mixed- on CT and MRI). In a tumor with septation, if the attenuation/intensity of different locules varied, the lesion was recorded as having a stained-glass appearance.

Discrepancy between reviewers and between imaging modalities were resolved by a third radiologist (K.K., with 19 years’ experience in abdominal radiology) who had the benefit of reviewing all available imaging modalities.

### Pathological findings

A board-certified pathologist reviewed gross and histologic analyses of all resected specimens and evaluated for the presence of invasive cancer and biliary prolapse as well as the evidence of ovarian type stroma (inclusion criterion).

### Statistical analyses

Statistical analysis was performed using R software (version 4.0.3, R Foundation for Statistical Computing, Vienna, Austria). We compared the clinical and radiologic features between MCN-L with and without biliary prolapse using the Mann–Whitney *U* test for continuous variables and the Fisher’s exact test for categorical variables. A *p* value of < 0.05 was considered to be significant. Comparison of each variable between readers was assessed by first-order agreement coefficient (AC1) using the Gwet method [19]. The interobserver agreement was defined as excellent (≥ 0.81), good (0.61–0.80), moderate (0.41–0.60), fair (0.21–0.4), and poor (≤ 0.20).

## Results

The median size of 34 MCN-Ls was 8.7 cm (range: 2.2–20.8 cm). Five MCN-Ls were located in the lateral section of the left lobe, 19 in the left medial section, and the remaining 10 in the right lobe. Table 1 shows the summary of all radiologic findings.

**Table 1:** Radiologic features of MCN-L

Image features	Frequency, ratio (%)		
Morphologic appearances			
Lobulation	8/34	24	
Thick cyst wall (> 2 mm)	21/34	62	
Cyst wall irregularity	25/34	74	
Cyst wall hemorrhage (MRI T1WI)	3/15	20	
Calcification of wall/septa (US/Precontrast CT)	4/21	19	
Septation (multilocularity)	33/34	97	
Cyst-in-cyst appearance	31/34	91	
Contrast-enhanced findings (DCE CT/MRI)			
Wall/septa enhancement	15/15	100	
Solid portion enhancement	1/15	7	
Intracystic content			
Cerebral spinal fluid-like (CT/MR) or anechoic (US) content	16/34		47
Stained glass appearance	13/34		38
Bile duct relationship			
Communication to the bile duct	0/34		0
Downstream bile duct dilation	0/34		0
Upstream bile duct dilation	17/34		50
Biliary prolapse	5/34		15

Data are number of cases with the imaging findings over number of cases evaluated, and frequency

*DCE CT* dynamic contrast-enhanced CT

### Clinical and imaging findings of MCN-L with and without biliary prolapse

**Fig. 1:**
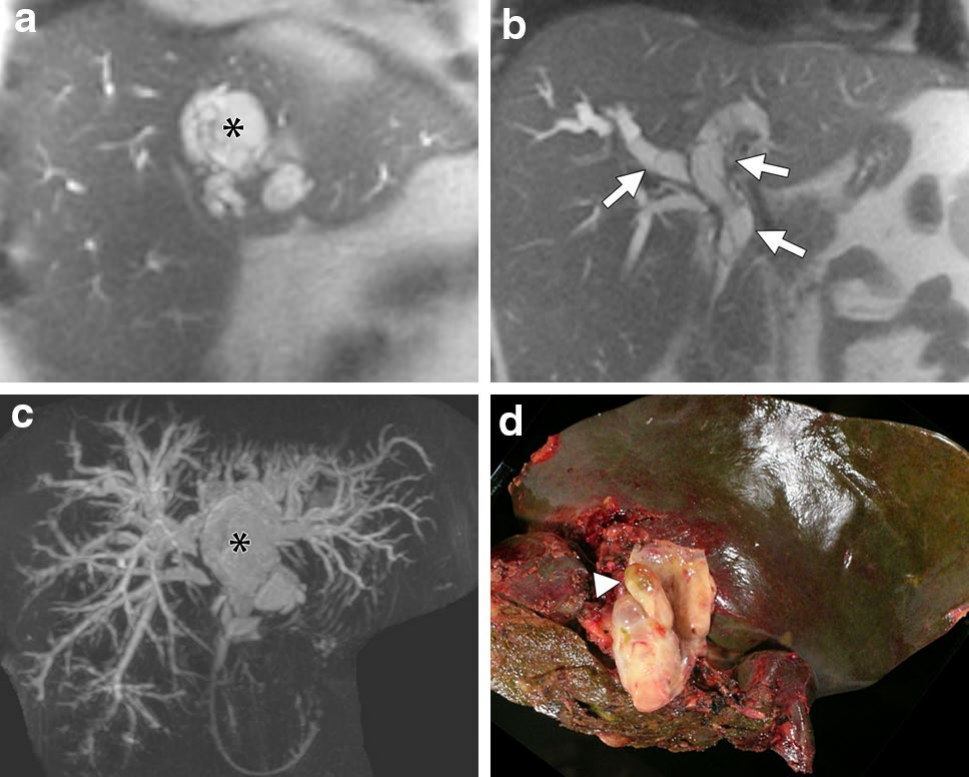
A 27-year-old woman, who presented with abdominal pain and jaundice, had a 63 mm multilocular cystic mass in segment IV of the left lobe of the liver (a, single shot fast spin echo coronal image. asterisk). Multilocular cystic mass also could be seen in both hepatic ducts extending to the extrahepatic duct, which indicated intraductal tumor prolapse (b, single shot fast spin echo coronal image. arrows). Peripheral bile duct dilation was also seen in MRCP (c, 2D-MRCP). The gross pathology picture demonstrates the biliary prolapse of the mucinous cystic neoplasm of the liver (d, gross pathology picture. arrowhead). Only MRI was available to review. Both readers could tell the biliary prolapse

**Fig. 2:**
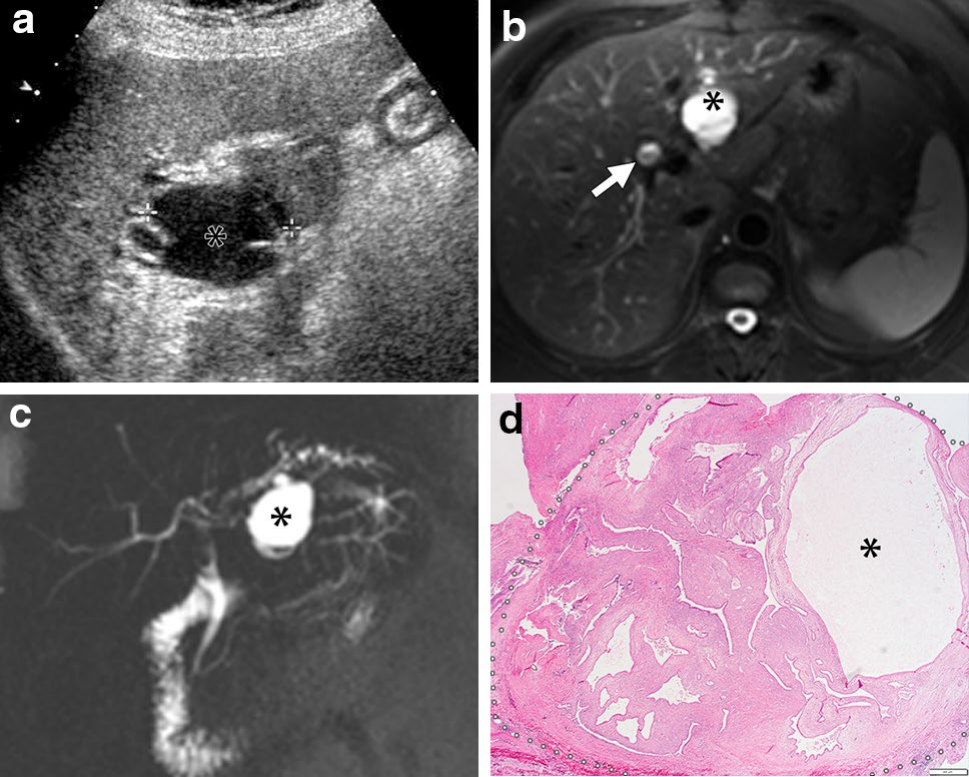
A 41-year-old woman presented with jaundice. A 35 mm multilocular cystic mass in the segment II of the liver was seen on US (a, asterisk) and MRI (b. fat-saturated T2 weighted image; c. MRCP). Biliary prolapse of the mass could be seen on common hepatic duct (b, arrow). Peripheral bile duct dilation was also seen in MRCP (c). Both readers could tell biliary prolapse on MRI, but not US. Microscopic specimen (d, H-E stain) showed the cystic tumor inside the biliary lumen (d, dashed line). Asterisk indicates a prominent cystic portion (d, asterisk)

Five of 34 MCN-Ls had biliary prolapse at pathology. Table 2 summarizes the clinical information of patients with MCN-L with biliary prolapse. Table 3 provides comparison of clinical information and imaging features of the MCN-Ls with and without biliary prolapse. On imaging, biliary prolapse was seen as well-demarcated multilocular cystic component protruding into the bile duct (Fig. 1, 2). The patients with biliary prolapse were significantly younger than those without prolapse (27 years [22–56] vs 51 years [14–82], effect size -0.37, *p* = 0.03), and the size of MCN-Ls with biliary prolapse was significantly smaller than that of MCN-Ls without biliary prolapse (6.4 cm [2.2–7.5] vs 9.6 cm [3.1–20.8], effect size − 0.43, *p* = 0.01). Jaundice was significantly more common in patients with biliary prolapse [80% (4/5) vs 3% (1/29), effect size 1.8, *p* = 0.0005]. There was no significant difference in the shape of the MCN-L between those with and without biliary prolapse (round/lobulated; 5/0 vs 21/8, *p* = 0.3). All MCN-Ls with biliary prolapse were associated with upstream bile duct dilation while 38% (11/29) MCN-Ls without biliary prolapse had upstream bile duct dilatation (effect size 1.8, *p* = 0.02) (Fig. 1). There was no biliary dilatation downstream to the tumor in MCN-L with or without biliary prolapse. There were no significant differences in other radiological features between MCN-L with and without biliary prolapse.

**Table 2:** Cases of MCN-L with biliary prolapse

Age	Gender	Image modality	Location	Size(cm)	Prolapse
27	F	MRI	S4	6.8	Left hepatic duct to common hepatic duct
56	F	MRI	S4	2.2	Left hepatic duct to common hepatic duct
22	F	CT and MRI	S6	6.4	Right hepatic duct to common hepatic duct
41	F	US and MRI	S3	3.5	Left hepatic duct
23	F	US and CT	S4	7.5	Left hepatic duct to common hepatic duct

**Table 3:** Comparison of radiologic and clinical features between MCN-L with and without biliary prolapse

	MCN with biliary prolapse *n* = 5	MCN without biliary prolapse *n* = 29	*p* value
Age, median years (range)	27 (22–56)	51 (14–82)	0.03
Size, median cm (range)	6.4 (2.2–7.5)	9.6 (3.1–20.8)	0.01
Signs/Symptom			
None	0	9 (31%)	0.3
Pain	3 (60%)	18 (62%)	1.0
Cholangitis	1 (20%)	0	0.1
Weight loss	0	1 (3%)	1.0
Jaundice	4 (80%)	1 (3%)	0.0005
Laboratory data			
Alkaline Phosphatase	286 (*n* = 1)	96.5 (44–217) (*n* = 13)	
Total bilirubin	7.2 (*n* = 1)	0.5 (0.1–1.5) (*n* = 11)	
Carbohydrate antigen 19–9	8 (*n* = 1)	214.5 (14–866) (*n* = 6)	
Carcinoembryonic antigen	NA	1.2 (0.7–4.1) (*n* = 5)	
Interval between imaging and surgery, median days (range)	37 (14–352)	38 (1–302)	0.81
Imaging features			
Location Right/Middle/Left*	1/3/1	9/16/4	0.3
Lobulation Yes/No	0/5	8/21	0.3
Septation Yes/No	5/0	28/1	1.0
Wall thickness Yes/No	2/3	20/9	0.3
Upstream bile duct dilatation Yes/No	5/0	11/18	0.02

*Right* right liver, *Middle* left medial section, *Left* left lateral section

Among 5 MCN-Ls with biliary prolapse, 4, 2, and 2 cases were examined by MRI/MRCP, CT, and US, respectively. The diagnostic accuracy of biliary prolapse in each reader in US, CT, and MRI were 0.882 and 0.941, 0.92 and 1.00, and 0.867 and 0.933, respectively. Specifically, of the 15 MRI examinations, all 4 cases with prolapse were correctly diagnosed by both readers, but there were two and one false positives by reader 1 and reader 2, respectively. Of the 25 CT examinations, one of two prolapse case was correctly diagnosed by reader 1 with one false positive; two of two prolapse cases were correctly diagnosed by reader 2 without false positive. Of the 17 US examinations, one of two prolapse case correctly diagnosed by both readers with one false positive by reader 1.

### Other radiological findings of MCN-L

**Fig. 3:**
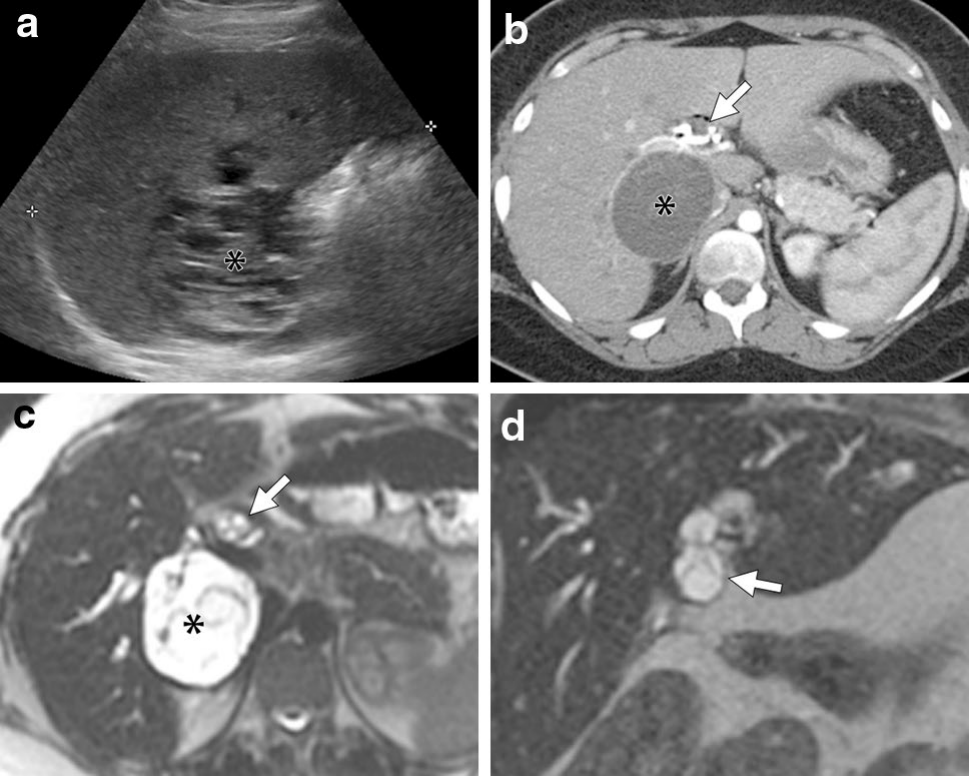
A 22-year-old woman presented with abdominal pain, fever, and jaundice. A 64 mm multilocular cystic mass in the segment VI of the liver was seen on US (a, asterisk), contrast-enhanced CT (b, asterisk), and MRI (c, single shot fast spin echo axial image, asterisk, d. single shot fast spin echo coronal image, asterisk). Arrows indicated the tumoral prolapse to the bile duct. Reader 2 could tell the biliary prolapse on US, CT and MRI, but reader 1 could tell it on US and MRI. Biliary prolapse was better delineated by MRI than CT because, as well as liver tumors, it showed separation (compare arrows in b and c). Abbreviation: GB, gallbladder

**Fig. 4:**
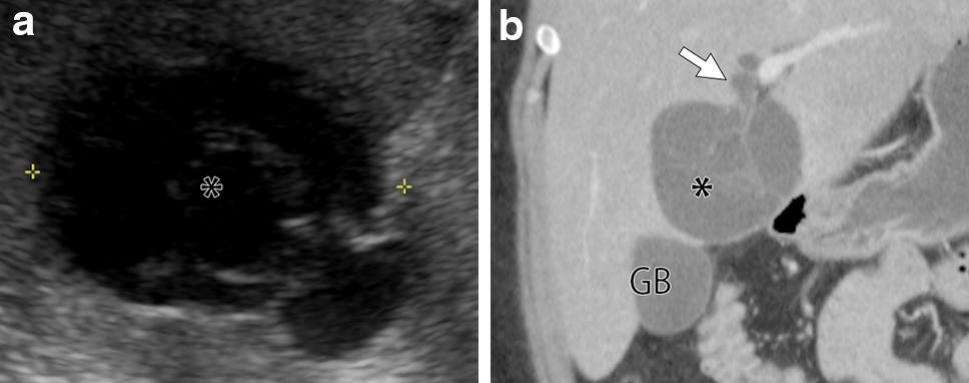
A 23-year-old woman presented with abdominal pain. She had a multilocular cystic mass 75 mm in diameter in the segment IV of the left lobe of the liver on US (a, asterisk) and contrast-enhanced CT (b, coronal image, asterisk). Arrows indicated the tumoral prolapse to the bile duct. Both readers could not tell the biliary prolapse on US, but could tell on CT. Abbreviation: GB, gallbladder

**Fig. 5:**
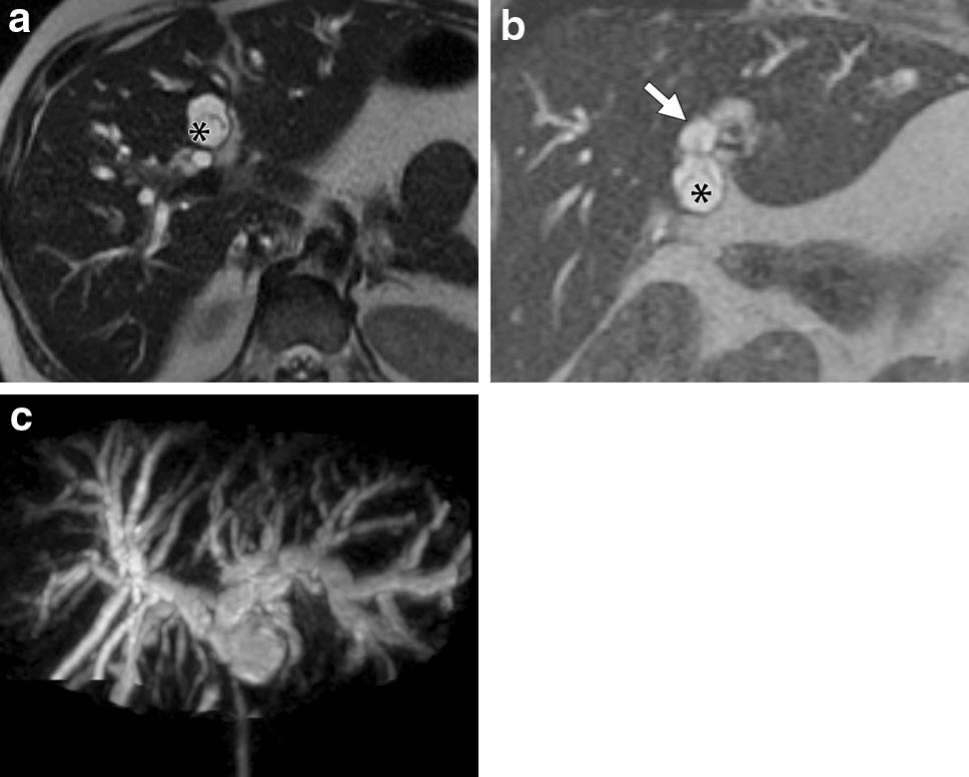
A 56-year-old woman presented with jaundice. She had a multilocular cystic mass 22 mm in diameter in the segment IV of the left lobe of the liver on MRI (a. single shot fast spin echo axial image, asterisk; b. single shot fast spin echo coronal image, asterisks; c. MRCP). Only MRI was available to review. Both readers could tell the biliary prolapse (b, arrow)

All MCN-Ls except for one (98%, 33 of 34 patients) had at least one intracystic septation resulting in a multilocular cystic mass (Figs. 1, 2, 3, 4 and 5). One MCN-L was unilocular cystic mass. A cyst-in-cyst appearance was seen in 91% (31 of 34) of the patients (Figs. 1, 2, 3, 4 and 5). The outer cyst wall was thick (> 2 mm thickness) in 21 MCN-Ls and was particularly prominent in two cases (Fig. 3). Wall hemorrhage was seen in 20% (3 of 15) MCN-Ls on T1 weighted images. Four of 21 (19%) had wall calcification.

In total, 15 patients had both pre and post-enhanced CT and/or MRI enabling assessment of the tumor wall and septa. All MCN-Ls had wall/septal enhancement (Fig. 3). Only one MCN-L (6.7%) had an enhancing solid portion. 21 of 34 (62%) MCN-Ls had homogeneous water-like fluid attenuation/signal intensity, and 13 of 34 (38%) MCN-Ls had heterogeneous attenuation/signal intensity (“stained-glass” appearance) (Fig. 4).

### Interobserver agreement of imaging findings of MCN-L by two radiologists

Interobserver agreement of two radiologists were shown in Table 4 (supplementary) with AC1 value, 95% confidence intervals, p value, and % agreement. Good to excellent agreement through modalities were seen in the assessment of septation (AC1 = 1.000, 0.955, and 1.000 in US, CT, and MRI), cyst-in-cyst appearance (AC1 = 0.816, 0.765, and 0.919 in US, CT, and MRI), and downstream bile duct dilation (AC1 = 1.000, 0.913, and 0.827 in US, CT, and MRI), and biliary prolapse (AC1 = 0.930, 0.906, and 0.627 in US, CT, and MRI). On the other hand, poor to fair agreements or variations among modalities were seen in the assessment of cyst wall thickness and irregularity (AC1 = 0.268, 0.258, and 0.608 in US, CT, and MRI), stained glass appearance (AC1 = 0.396, 0.943, and 0.600 in US, CT, and MRI), enhanced solid portion (AC1 = 0.647, 0.461 in CT and MRI), intracystic contents (AC1 = 0.632, 0.496, and − 0.026 in US, CT, and MRI), and upstream bile duct dilation (AC1 = − 0.006, 0.444, and 0.869 in US, CT, and MRI).

### Pathologic appearances

All MCN-Ls had ovarian-like stroma in the wall (inclusion criterion). None of MCN-Ls in this study were associated with invasive carcinoma. In one case, an enhancing solid portion was granulation tissue without any atypical cells. In cases of MCN-L with biliary prolapse, the prolapsed portion of the lesion was distinct from the bile duct lumen by the wall of cystic tumor and no rupture of cystic wall was seen.

## Discussion

Prolapse of the tumor into the bile duct was observed in 15% (5 of 34) of patients with MCN-Ls in this study. This prevalence may be overestimated because MCN-L without biliary prolapse is less symptomatic and may form an undiagnosed population. Choi et al. reported a case of biliary prolapse in a series of 8 cases of biliary cystadenoma almost 30 years ago [14]. The prevalence of BP in Chois' study was 12.5% (1/8) and it is similar to our study, although presence or absence of OLS was not reported in their study. To the best of our knowledge, there are no reports of other cystic liver tumors or non-neoplastic cystic liver lesions showing biliary prolapse [5, 20–22]. We consider this finding is specific to MCN-L and thus helpful in making the correct diagnosis of MCN-L.

Our study showed that biliary prolapse in MCN-L occurs in relatively younger female (median 27 years [22–56] in MCN-L with biliary prolapse vs. 51 years [14–82] in MCN-L without biliary prolapse, *p* = 0.03) and relatively smaller tumor (median size 6.4 cm [2.2–7.5] in MCN-L with biliary prolapse vs. 9.6 cm [3.1–20.8] in MCN-L without biliary prolapse, *p* = 0.01). To date, 16 cases of biliary prolapse of MCN-L have been reported in the radiology literature in either case reports or small case series [11–13] (supplemental Table 2). The median age and size of these 15 cases were 37.5 years [20–62] and 5.8 cm [2.8–10], respectively, and were similar to our study results of MCN-L with biliary prolapse. Zen et al. reported 54 MCN-Ls in a pathology literature, which is the largest series to date [6]. The median age and size of their cases were 53 years [21–80] and 10.0 cm [2.9–24.0]. Although they did not mention the biliary prolapse, majority of them were probably without biliary prolapse and their results were similar to our study results of MCN-L without biliary prolapse.

Our study showed that MCN-L with biliary prolapse commonly presented with jaundice (80%, 4/5 vs. 3%, 1/29, *p* = 0.0005) and showed upstream bile duct dilation (100%, 5/5 vs. 38%, 11/29, *p* = 0.02). Of the previously reported MCN-Ls with biliary prolapse, clinical symptom was available in 14 cases and in such cases, all cases but one presented with jaundice and with and without other various symptom such as pain and fever [11–13]. On the other hand, among case series of MCN-L which described symptom of the patients, only 27% (4 of 15) and 33% (3 of 9) were symptomatic [4, 5].

MCN-L predominantly involved the left lobe of the liver regardless of presence or absence of biliary prolapse. This is concordant with previous studies [5, 6, 11].

MRI/MRCP was the most common imaging modality used in patients with MCN-L with biliary prolapse in this study, presumably due to the presenting symptom of jaundice. Identification of cyst wall or septations extending into the bile duct is crucial for making the diagnosis of prolapse, however, the appearance of biliary prolapse can be subtle [11, 13]. To detect prolapsed lesions correctly, it is essential to depict the thin septa or wall in the bile ducts (Figs. 1, 3, 5). Therefore, images with excellent contrast and spatial resolution are important to improve the diagnostic accuracy. From this point of view, high spatial T2 weighted images including 3-dimensional MRCP and single shot fast spin echo sequences are considered to be the most desirable non-invasive examination method (Figs. 1, 2, 3, 5). In our study, the accuracy of MRI/MRCP in detecting biliary prolapse was excellent for both readers (87% for reader 1 and 93% for reader 2). It is considered that the cause of the false positive for biliary prolapse in MRI/MRCP was due to the biliary compression by the cystic tumor and septate locules near the compressed site, which mimicked biliary prolapse. Thus, it may be important to carefully examine the bile duct especially when a cystic mass causes intrahepatic bile duct dilation since all of MCN-L with biliary prolapse showed upstream bile duct dilation. ERCP is also useful for delineating biliary prolapse by filling defects in the extrahepatic bile ducts, but it cannot estimate the bile ducts distal to the tumor, while MRCP is useful in assessing the extent of the lesion and can provide a road map of the bile ducts proximal to the lesion prior to surgical intervention. ERCP offers the possibility to relief of jaundice by insertion of endobiliary drainage tube or stent [23, 24].

It is interesting the differentiation between biliary prolapse from biliary invasion. The biliary prolapse of MCN-L is not invasion but just protrusion of the cystic component to intraluminal of bile duct. In fact, there was no invasive carcinoma in the reported MCN-L with biliary prolapse (supplemental table) nor in our cohort. Given the preference for locations close to the hepatic hilum (more common in S4) of MCN-L with biliary prolapse, tumors arising from this site may be prone to prolapse into the bile duct because their proximity to the relatively thick central side of the bile duct, as hypothesized by Takano et al. [11].

Cyst forming intraductal papillary neoplasm of the bile duct (so-called cystic IPNB) is one of the main differential diagnoses [4, 5, 21]. The characteristic imaging features of cystic IPNB include cyst communicating with the bile duct and downstream bile duct dilatation due to abundant production mucin [4, 21]. Mural nodule is commonly present (15/16 in Kim’s study and 10/10 in Zen’s study) [3, 4]. Septation is also commonly seen, and Kim et al. reported that the centrally located septation rather than peripheral septation is a characteristic finding of cystic IPNB compared to MCN-L [4]. In our study, none of the MCN-L showed communication with the bile duct or dilation of downstream bile duct. Enhanced solid portion was seen in 1 patient which was due to granulation tissue. IPNB commonly contains invasive carcinoma, while MCN-L rarely does [5, 6].

In our study, none of MCN-Ls had invasive carcinoma. However, we could not compare the imaging findings between cystic IPNB and MCN-L because cystic IPNB is rare in the western countries, and only 1 case was identified in the surgical database at our institution.

Mixed epithelial and stromal tumors of the kidney [25] are known to show propensity to prolapse into the renal collecting system. Interestingly, mixed epithelial and stromal tumors contain ovarian-like stroma. MCN-L is thought to be a counterpart to the pancreatic MCN [26], but to our knowledge there is no report of MCN of the pancreas prolapsing into the pancreatic duct. The mechanism of biliary prolapse in MCN-L remains to be clarified.

Our study had several limitations. First, we did not compare MCN-Ls with other biliary cystic neoplasms such as cystic IPNB, other cystic liver tumors without OLS or benign bile duct cysts. However, based on our experience and previous reports, biliary prolapse of a cystic neoplasm is seen exclusively in MCN-L. Second, our study cohort included a relatively small number of patients. Further studies with larger patient populations are required. Third, because this was a retrospective study, the ultrasound, CT, and MRI examinations were performed on a variety of machines using nonstandardized technical parameters. The lack of standardization of imaging parameters could affect image analysis.

In conclusion, the frequency of MCN-L with biliary prolapse was 15% in our study. It commonly occurred in younger female patients, and the tumor tended to be smaller than MCN-L without biliary prolapse. Jaundice and peripheral bile duct dilatation were common.

## Supplementary Information

Below is the link to the electronic supplementary material.

Supplementary file1 (DOCX 24 KB)

Supplementary file2 Biliary prolapse of MCN-L: literature cases (DOCX 31 KB)

## Data Availability

Nothing special to mention.

## Abbreviations

MCN-L: Mucinous cystic neoplasm of the liver
IPNB: Intraductal papillary mucinous neoplasm of the bile duct
OLS: Ovarian-like stroma
WHO: World Health Organization
