# Supplementary material for: Mucinous cystic neoplasms of the liver with biliary prolapse
Source: Jpn J Radiol. 2022 Nov 19;41(4):409–16. doi: 10.1007/s11604-022-01361-3 (PMC10066120; doi:10.1007/s11604-022-01361-3)
Supplement: Supplementary file 1 — Supplementary file1 (DOCX 24 KB) [file 11604_2022_1361_MOESM1_ESM.docx]

## Table 4 (Supplementary table). Interobserver agreement of two radiologists in each finding

| US / Radiologic features | n | R1 | R2 | AC1 | 95%CI | p value | % agreement |
| --- | --- | --- | --- | --- | --- | --- | --- |
| Bile duct relationship |  |  |  |  |  |  |  |
| Bile duct communication | 17 | 0 | 0 | 1.000 | (NaN) | NaN | 100% |
| downstream bile duct dilation | 12 | 1 | 1 | 1.000 | (1,1) | <0.001 | 100% |
| upstream bile duct dilation | 17 | 10 | 0 | -0.006 | (-0.637,0.625) | 1.0 | 41% |
| biliary prolapse | 17 | 2 | 1 | 0.930 | (0.773,1) | <0.001 | 94% |
|  |  |  |  |  |  |  |  |
| Morphologic appearance |  |  |  |  |  |  |  |
| Lobulation | 17 | 7 | 5 | 0.477 | (-0.009,0.963) | 0.05 | 71% |
| Thick cyst wall | 17 | 13 | 10 | 0.268 | (-0.289,0.824) | 0.3 | 59% |
| Cyst wall irregularity | 17 | 16 | 6 | -0.083 | (-0.688,0.523) | 0.8 | 41% |
| Calcification of wall/septa | 17 | 0 | 1 | 0.938 | (0.798,1) | <0.001 | 94% |
| Septation | 17 | 16 | 16 | 1.000 | (1,1) | <0.001 | 100% |
| Cyst-in-cyst appearance | 17 | 13 | 13 | 0.816 | (0.527,1) | <0.001 | 88% |
|  |  |  |  |  |  |  |  |
| Intracystic content |  |  |  |  |  |  |  |
| anechoic content | 17 | 8 | 5 | 0.443 | (-0.046,0.932) | 0.07 | 71% |
| stained glass appearance | 17 | 7 | 3 | 0.396 | (-0.124,0.917) | 0.1 | 65% |
|  |  |  |  |  |  |  |  |
| CT / Radiologic features | n | R1 | R2 | AC1 | 95%CI | p value | % agreement |
| Bile duct relationship |  |  |  |  |  |  |  |
| Bile duct communication | 25 | 0 | 0 | 1.000 | (NaN) | NaN | 100% |
| downstream bile duct dilation | 25 | 2 | 0 | 0.913 | (0.78,1) | <0.001 | 92% |
| upstream bile duct dilation | 25 | 14 | 10 | 0.444 | (0.064,0.823) | 0.02 | 72% |
| biliary prolapse | 25 | 2 | 1 | 0.906 | (0.762,1) | <0.001 | 92% |
|  |  |  |  |  |  |  |  |
| Morphologic appearance |  |  |  |  |  |  |  |
| Lobulation | 25 | 7 | 14 | 0.077 | (-0.368,0.522) | 0.7 | 52% |
| Thick cyst wall | 25 | 21 | 11 | 0.258 | (-0.176,0.693) | 0.2 | 60% |
| Cyst wall irregularity | 25 | 19 | 5 | -0.192 | (-0.613,0.229) | 0.4 | 40% |
| Calcification of wall/septa | 5 | 3 | 2 | 0.600 | (-0.511,1) | 0.2 | 80% |
| Septation | 25 | 24 | 23 | 0.955 | (0.858,1) | <0.001 | 96% |
| Cyst-in-cyst appearance | 25 | 20 | 18 | 0.765 | (0.508,1) | <0.001 | 84% |
|  |  |  |  |  |  |  |  |
| Contrast-enhanced findings |  |  |  |  |  |  |  |
| Wall/septa enhancement | 4 | 4 | 4 | 1.000 | (NaN) | NaN | 100% |
| Solid portion enhancement | 4 | 2 | 0 | 0.647 | (-1.858,1) | 0.8 | 50% |
|  |  |  |  |  |  |  |  |
| Intracystic content |  |  |  |  |  |  |  |
| cerebral spinal fluid-like content | 25 | 5 | 6 | 0.943 | (0.822,1) | <0.001 | 96% |
| stained glass appearance | 25 | 5 | 5 | 0.943 | (0.822,1) | <0.001 | 96% |
|  |  |  |  |  |  |  |  |
| MRI / Radiologic features | n | R1 | R2 | AC1 | 95%CI | p value | % agreement |
| Bile duct relationship |  |  |  |  |  |  |  |
| Bile duct communication | 15 | 0 | 0 | 1.000 | (NaN) | NaN | 100% |
| downstream bile duct dilation | 15 | 3 | 1 | 0.827 | (0.542,1) | 0.000 | 87% |
| upstream bile duct dilation | 15 | 7 | 6 | 0.869 | (0.587,1) | 0.000 | 93% |
| biliary prolapse | 15 | 5 | 5 | 0.627 | (0.175,1) | 0.001 | 80% |
|  |  |  |  |  |  |  |  |
| Morphologic appearance |  |  |  |  |  |  |  |
| Lobulation | 15 | 2 | 5 | 0.481 | (-0.047,1) | 0.071 | 67% |
| Thick cyst wall | 15 | 14 | 10 | 0.608 | (0.153,1) | 0.012 | 73% |
| Cyst wall irregularity | 15 | 10 | 6 | 0.469 | (-0.038,0.977) | 0.067 | 73% |
| Cyst wall hemorrhage | 15 | 3 | 3 | 0.804 | (0.487,1) | 0.000 | 87% |
| Septation | 15 | 14 | 14 | 1.000 | (1,1) | 0.000 | 100% |
| Cyst-in-cyst appearance | 15 | 14 | 13 | 0.919 | (0.733,1) | 0.000 | 93% |
|  |  |  |  |  |  |  |  |
| Contrast-enhanced findings |  |  |  |  |  |  |  |
| Wall/septa enhancement | 12 | 12 | 12 | 1.000 | (NaN) | NaN | 100% |
| Solid portion enhancement | 12 | 3 | 0 | 0.680 | (0.224,1) | 0.007 | 75% |
|  |  |  |  |  |  |  |  |
| Intracystic content |  |  |  |  |  |  |  |
| cerebral spinal fluid-like content | 15 | 8 | 5 | 0.607 | (0.15,1) | 0.013 | 80% |
| stained glass appearance | 15 | 9 | 6 | 0.600 | (0.141,1) | 0.014 | 80% |

R1, Reader 1; R2, Reader 2; AC1, first-order agreement coefficient; CI, confidence interval
