# Supplementary material for: Mucinous cystic neoplasms of the liver with biliary prolapse
Source: Jpn J Radiol. 2022 Nov 19;41(4):409–16. doi: 10.1007/s11604-022-01361-3 (PMC10066120; doi:10.1007/s11604-022-01361-3)
Supplement: Supplementary file 2 — Supplementary file2 Biliary prolapse of MCN-L: literature cases (DOCX 31 KB) [file 11604_2022_1361_MOESM2_ESM.docx]

Supplemental table 2

| Ref | Age | Sex | Symptoms | Size (mm) | Primary site of the tumor | Histology |
| --- | --- | --- | --- | --- | --- | --- |
| 1 | 61 | F | NA* | 100 x 80 | segment 4B | cystadenoma |
| 1 | 38 | F | NA* | 100 x 90 | segment 4B | cystadenoma |
| 2 | 32 | F | upper abdominal pain, jaundice, and diarrhea | 79 x 76 x 65 | segment 3 | cystadenoma |
| 3 | 25 | F | right hypochondrial pain and episodic biliary obstruction | 55 x 40 | segment 4B to 3 | cystadenoma |
| 4 | 56 | F | right hypochondrial pain and spontaneously remitted jaundice | 55 | segment 4 | cystadenoma |
| 5 | 34 | F | jaundice (recurrent episode) | 45 x 40 | segment 4 | cystadenoma |
| 6 | 57 | F | epigastric and right hypochondriac pain and jaundice | 50 x 45 | segment 4 | cystadenoma |
| 7 | 28 | F | upper abdominal pain | 73 x 70 | segment 4 | cystadenoma |
| 8 | 62 | F | dysuria and hyperpigmentation of urine | NA* | segment 4 | cystadenoma |
| 9 | 41 | F | epigastric pain | 30 x 16 | segment 4B to 3 | cystadenoma |
| 10 | 39 | F | jaundice | NA* | segment 4 | cystadenoma |
| 11 | 37 | F | intermittent abdominal bloating and discomfort for 20 months | 29 x 24 | segment 4 | cystadenoma |
| 12 | 57 | F | sudden abdominal pain and fever | 83 x 80 | segment 4 | MCN with low grade dysplasia |
| 12 | 26 | F | jaundice | 61 x 39 | segment 4 | MCN with low grade dysplasia |
| 13 | 20 | F | intermittent upper abdominal pain, jaundice, fever, and pruritus | 60 x 50 | segment 4B | MCN** |
| 13 | 28 | F | jaundice, pruritus, and anorexia | 28 x 14 | segment 4B | MCN** |

*: No specific description was noted.

**: No invasive or carcinoma was noted.

(1-13)

1. Gadzijev EM, Pleskovic A, Stanisavljevic D, Ferlan-Marolt V, Trotovsek B. Hepatobiliary cystadenoma can protrude and grow into the bile ducts. Hepatogastroenterology 1998;45:1446-1451

2. Gonzalez M, Majno P, Terraz S, Morel P, Rubbia-Brandt L, Mentha G. Biliary cystadenoma revealed by obstructive jaundice. Dig Liver Dis 2009;41:e11-13

3. Siriwardana PN, Pathirana A. Episodic biliary obstruction due to an intrahepatic biliary cystadenoma: A case report. J Med Case Reports 2009;3:9032

4. Yi B, Cheng QB, Jiang XQ, Liu C, Luo XJ, Dong H et al. A special growth manner of intrahepatic biliary cystadenoma. World J Gastroenterol 2009;15:6134-6136

5. Saravanan MN, Singh B, Ravindranath K, Raghavendra RR. Episodic jaundice due to an intrahepatic biliary cystadenoma with biliary stricture masquerading as hydatid cyst. Trop Gastroenterol 2010;31:332-335

6. Harmouch T, Vullierme MP, Sauvanet A, Paradis V, Amarti A. Hepatobiliary cystadenoma revealed by a jaundice: A case report. Case Rep Gastrointest Med 2011;2011:895605

7. Abe Y, Kasuya K, Itoi T, Kikuchi S, Ikeuchi N, Ishii K et al. Hepatobiliary cystadenoma of the liver prolapsing into the extrahepatic bile duct (with video). Gastrointest Endosc 2012;75:1099; discussion 1099-1100

8. Soochan D, Keough V, Wanless I, Molinari M. Intra and extra-hepatic cystadenoma of the biliary duct. Review of literature and radiological and pathological characteristics of a very rare case. BMJ Case Rep 2012;2012

9. Vyas S, Markar S, Ezzat T, Rodriguez-Justo M, Webster G, Imber C et al. Hepato-biliary cystadenoma with intraductal extension: Unusual cause of obstructive jaundice. Journal of gastrointestinal cancer 2012;43 Suppl 1:S32-37

10. Chandrasinghe PC, Liyanage C, Deen KI, Wijesuriya SR. Obstructive jaundice caused by a biliary mucinous cystadenoma in a woman: A case report. J Med Case Rep 2013;7:278

11. Rayapudi K, Schmitt T, Olyaee M. Filling defect on ercp: Biliary cystadenoma, a rare tumor. Case Rep Gastroenterol 2013;7:7-13

12. Takano Y, Nagahama M, Yamamura E, Maruoka N, Mizukami H, Tanaka J et al. Prolapse into the bile duct and expansive growth is characteristic behavior of mucinous cystic neoplasm of the liver: Report of two cases and review of the literature. Clin J Gastroenterol 2015;8:148-155

13. Anand S, Chandrasekar S, Raja K, Pottakkat B. Mucinous cystic neoplasm of the liver with biliary communication: An exception to the current classification. BMJ Case Rep 2019;12
